# Small but mighty: the causes and consequences of micronucleus rupture

**DOI:** 10.1038/s12276-020-00529-z

**Published:** 2020-11-24

**Authors:** Mijung Kwon, Mitchell L. Leibowitz, Jae-Ho Lee

**Affiliations:** 1 Department of Life Science and the Research Center for Cellular Homeostasis, Ewha Womans University, Seoul, 03760 Korea; 2 Department of Pediatric Oncology, Dana-Farber Cancer Institute, Boston, MA USA; 3 Department of Cell Biology, Harvard Medical School, Boston, MA USA; 4 Department of Biochemistry and Molecular Biology, Suwon, 16499 South Korea; 5 Institute of Medical Science, Ajou University School of Medicine, Suwon, 16499 South Korea

**Keywords:** Cell biology, Cancer

## Abstract

Micronuclei are small DNA-containing nuclear structures that are spatially isolated from the main nucleus. They are frequently found in pathologies, including cancer. It was recently shown that these nuclear structures are not only biomarkers of disease but also play an active role in tumor biology. Many consequences of micronucleus formation on tumor biology are dependent on the frequent and irreversible rupture of their nuclear envelopes, which results in the exposure of their DNA contents to the cytoplasm. In this review, we discuss models of defective nuclear envelope deposition on missegregated chromosomes that lead to nuclear envelope rupture. Furthermore, we expound upon the various downstream consequences of micronucleus nuclear envelope rupture on cells. These consequences include a massive DNA rearrangement phenomenon called chromothripsis and activation of the cGAS-STING innate immune signaling pathway, which can be a double-edged sword with tumorigenesis and tumor prevention functions. Although micronuclei are small structures, the impact they have on cells and their microenvironment is quite large.

## Cancer: Rupture of micronuclei drives genome instability

Micronuclei, which contain faulty chromosomes or chromosome fragments and occur outside the main cellular nucleus, are prone to rupturing, which leads to DNA changes that can drive tumor development. A team led by Mijung Kwon from Ewha Womans University in Seoul and Jae-Ho Lee of Ajou University School of Medicine in Suwon, both in South Korea, review how these micronuclei tend to burst, spilling their contents into the cell with devastating consequences. The chromosomes they contain break into tiny fragments and this broken DNA finds its way into the main nucleus, leading to chromosomal rearrangements that can permanently alter genomic function. The rupture of micronuclei also activates a part of the innate immune system that can promote cancer cell invasion and spread. Drugs targeting these processes could aid in the treatment of cancer.

## An old observation with a new meaning

**Fig. 1:**
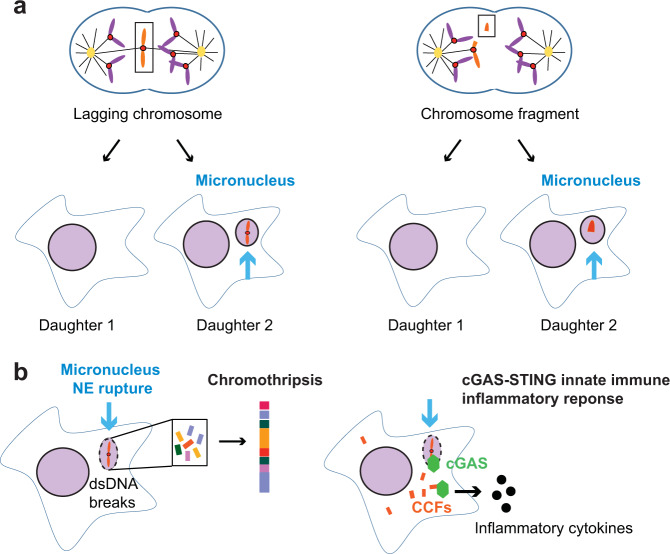
Micronucleus formation and its downstream consequences. **a** Micronuclei form from lagging chromosomes (left) or chromosome fragments (right) following mitotic errors or DNA damage, respectively. Although micronuclei are enclosed by NE, their NE is fragile, leading to catastrophic NE rupture. **b** (Left) Chromosomes contained in micronuclei with a ruptured NE acquire double-strand (ds) DNA breaks and chromosome pulverization, leading to chromothripsis, a phenomenon of extensive chromosome rearrangements confined to one or a few chromosomes. (Right) Chromatin released into the cytosol by NE rupture is recognized by cGAS, triggering the activation of cGAS-STING-dependent innate immune signaling. Cytoplasmic chromatin fragments (CCFs), which can also cause a cGAS-STING-dependent innate immune response, can be generated by autophagic degradation of the main nucleus or from DNA fragments from a micronucleus.

At mitotic exit, extensive nuclear envelope (NE) remodeling ensures that separated chromosomes are enclosed by a NE and that a single nucleus is formed in each daughter cell^1,2^. A micronucleus, however, is an isolated nuclear structure separated from the main nucleus and represents an extreme example of nuclear atypia. Micronuclei arise from lagging chromosomes or chromosome fragments caused by mitotic errors or DNA damage^3–6^ (Fig. 1a). They have long been used as biomarkers of genotoxicity, tumor risk, and tumor grade^5–9^. The frequent observation of micronuclei in tumors and after genotoxic events raises the specter that micronuclei might not merely be passenger events but might play active roles in DNA damage and tumor progression.

The study of micronuclei and their potential role in DNA damage dates back to at least 1968. In this seminal work, Kato and Sandberg proposed that micronuclei are associated with chromosome pulverization, a visual phenomenon of apparent discontinuous DNA fragments on chromosome spreads^10^. This was followed by the observation that premature chromatin condensation and a pulverized appearance of chromosomes can be induced in the nuclei of under-replicated S-phase cells upon their fusion with mitotic cells^11,12^. Despite the initial hypothesis that pulverized chromosomes might represent broken DNA, it was concluded that the pulverized appearance is merely the aberrant condensation of a chromosome that lagged during the cell cycle. With this finding, the field was largely forgotten for decades.

Only recently has the idea that micronuclei might lead to DNA damage and disease been revived (Fig. 1b). First, cytological evidence demonstrated that micronuclei undergo delayed DNA replication and DNA damage^9,12–14^. Later it was shown that the NE of micronuclei is fragile and ruptures, leading to the loss of nuclear-cytoplasmic compartmentalization^15^. This rupture originates from defective postmitotic NE assembly that persists into interphase^16^ and results in the release of micronuclear contents into the cytosol^15^. These steps then lead to DNA damage and chromosome pulverization^12^ (Fig. 1b, left). More recently, it was revealed that the DNA from ruptured micronuclei can also act as a source of cytoplasmic DNA and trigger an innate immune proinflammatory response^17–19^, which is frequently seen in cancer (Fig. 1b, right). Thus, micronuclei contribute broadly to many aspects of cancer biology.

## Built to fail: defective micronuclear envelope assembly

Many of the downstream consequences of micronucleus formation are intricately associated with micronuclear NE fragility^15^. Why micronuclei have fragile NEs and how NE rupture occurs are key questions that require further exploration. Interestingly, micronuclear NE fragility is known to originate in the prior mitosis^16^. To better understand the reasons for NE fragility, we first discuss normal NE assembly.

In higher eukaryotic cells that undergo open mitoses, the NE disassembles during each cell division, enabling mitotic spindle assembly. During mitotic exit the nuclear boundary is reestablished through the complex process of postmitotic NE reassembly^1,2^. NE reassembly requires inactivation of mitotic kinases and activation of protein phosphatases that reverse the mitotic phosphorylation of NE proteins and components of the nuclear pore complex (NPC)^1,2^. In addition, the concerted action of the NPC assembly factor ELYS and the RanGTP gradient on chromosomes promotes the recruitment of NE and NPC proteins to chromatin^20–22^. NE reformation around the chromosome mass is further promoted by the recruitment and expansion of the mitotic ER membrane to chromatin^23^. Finally, the microtubule-severing protein spastin and endosomal sorting complex required for transport-III (ESCRT-III) promote the disassembly of spindle microtubules and seal the NE holes that microtubules leave behind^24,25^. Thus, multiple mechanisms cooperate to ensure the formation of a functional NE barrier with NPCs, enabling efficient nuclear cytoplasmic transport.

**Fig. 2:**
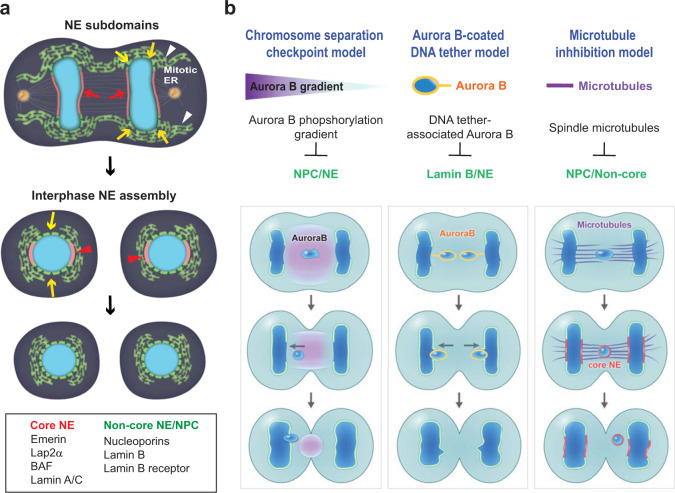
NE reassembly at mitotic exit and three proposed models to explain micronuclear NE defects. **a** (Top) Transient partitioning of NE domains during telophase: non-core proteins/NPCs are recruited to the chromosome periphery forming a non-core NE domain (yellow arrows); core NE proteins are recruited to chromosome regions facing spindle microtubules forming a core NE domain (red arrows). (Middle) During interphase, NPC-depleted pore-free islands (red arrowheads) are populated by NPCs and other non-core NE proteins (yellow arrows), resulting in a completely assembled and functional NE (bottom). Adapted from Liu et al.^16^. **b** Three models proposed to explain defective NE assembly on lagging chromosomes and micronuclei. For each model, proposed mechanisms inhibiting NE assembly on lagging chromosomes are indicated by NE and NPC components that were used in each study. For the Aurora B-dependent checkpoint and Aurora B-coated DNA tether models, core NE proteins were not assessed for NE assembly in the original studies.

Despite the mechanisms to facilitate concurrent recruitment of NE and NPC proteins^1,2^, initial post-mitotic NE reassembly displays a partitioning of NE domains that are largely depleted of or enriched with NPCs^26–29^ (Fig. 2a). Chromosome regions far from spindle microtubules assemble NPC-rich NEs by recruiting non-core NE proteins, which include NPCs, lamin B and lamin B receptor (LBR)^30,31^. This process is distinct from that of chromosome regions near dense spindle microtubules, which assemble NEs that lack NPCs but are enriched with core NE proteins such as barrier-to-autointegration factor (BAF), emerin, lamin-associated peptide 2α (LAP2α) and lamin A/C^26–28,30–33^. As a consequence of this separation, the NPC-depleted core domains display NPC gaps, called “pore-free islands”^29,33,34^. These gaps are later filled by incorporation of NPC into pore-free islands during interphase, resulting in the formation of a closed NE capable of functional nuclear-cytoplasmic transport^29,34^.

Given this highly coordinated model of NE assembly after mitosis, the reasons for micronuclei fragility and whether micronuclei follow similar spatiotemporal patterns of NE and NPC assembly have been areas of intense research. Although it is appealing to think that micronuclei might start with a normal NE prior to spontaneous NE disruption and loss of lamina continuity^15^, defects in NPC density and function in micronuclei have long been suggested^12,35,36^. Accordingly, many groups have focused on investigating postmitotic reassembly of NE on missegregated chromosomes^16,37–41^ (Fig. 2b). Despite some differences in the experimental systems used and conclusions drawn from these studies, it is now clear that micronuclei are initially generated with an altered composition of NE and NPC proteins.

One mechanism to explain NE defects in micronuclei is an Aurora B-dependent chromosome separation checkpoint model^37,42^ (Fig. 2b, left). This model proposes that a phosphorylation gradient of the mitotic kinase Aurora B monitors the position of chromosomes and inhibits NE assembly. In this model, high Aurora B activity at the spindle midzone senses lagging chromosomes and prevents NE assembly to ensure that membrane-free lagging chromosomes can be reincorporated into the main nucleus. This conclusion is drawn from the observation in *Drosophila* S2 cells that lagging chromosomes fail to recruit lamin B and some NPC components, and Aurora B inhibition reverses this NE defect^37^. Defective NE assembly was inferred in this study by the observation of mainly non-core NE proteins, leaving open the possibility that core NE proteins were being properly recruited to lagging chromosomes even in the presence of high Aurora B activity at the midzone. Later experiments by the same group suggested that Aurora B and CDK1 cooperate in the checkpoint—that Aurora B sets a CDK1 phosphorylation gradient by stabilizing Cyclin B and thus globally inhibits NE assembly of lagging chromosomes^38^.

Another group proposed a model in which Aurora B kinase inhibits NE assembly by a somewhat different mechanism that does not involve a checkpoint. By examining lagging acentric chromosomes in *Drosophila* neuroblasts, Karg et al. propose that a localized pool of Aurora B prevents NE assembly around acentric fragments and the DNA tethers that connect the fragments to their centric partners^39^ (Fig. 2b, middle). The Aurora B-coated DNA tether model is based on the observation that lamin B gaps colocalize with DNA tethers coated with Aurora B and its substrate, phosphorylated histone H3. Moreover, the authors demonstrate that Aurora B depletion restores lamin B continuity^39^. A later study by the same group further suggests that a failure to recruit heterochromatin protein 1 (HP1a) to lagging acentric fragments causes a delay in NE assembly, possibly owing to the ability of HP1a to interact with LBR^40^.

Polo-like kinase 1 (Plk1) has also been proposed to contribute to postmitotic NE assembly^41^. Although lagging chromosomes can recruit some nuclear proteins (lamin A/C), they are inefficient at recruiting NPC proteins in HeLa cells, suggesting that there might be an independent regulatory mechanism that controls different NE and NPC proteins^41^. In search for such regulatory mechanisms, the role of Plk1 was explored. From these experiments it was revealed that inhibition of Plk1 only partially restored NPC assembly defects on lagging chromosomes^41^, raising the possibility that additional factors in NE assembly remained undiscovered.

To this end, Liu and Kwon et al. conducted a systematic examination of the recruitment of different NE and NPC proteins to lagging chromosomes in human cells^16^. They found that despite normal recruitment of core NE proteins and A-type lamins, non-core NE proteins, including NPCs and B-type lamins, were not recruited to lagging chromosomes^16^ (Fig. 2b, right). This NPC assembly defect on lagging chromosomes coincided with defective nuclear import, indicating it is likely a major contributing factor to NE disruption^16^. Moreover, by using correlative light and electron microscopy (CLEM) to directly visualize membrane assembly on lagging chromosomes, Liu and Kwon et al. found that lagging chromosomes assemble their NE without significant delay relative to NE assembly on the main nucleus^16^. These findings are unlike what would be expected in the presence of a chromosome segregation checkpoint^37,42^ wherein NE assembly on lagging chromosomes would be turned off in a switch-like manner. Instead, these findings suggest that defective core-only NE assembly on lagging chromosomes is an unfortunate accident of NE assembly.

But what causes core-only NE assembly on micronuclei? A particularly telling observation came from how NE assembly occurs around the main chromosome mass. Liu and Kwon et al. showed that NPCs and non-core NE proteins are mainly excluded from chromosome regions that face the spindle but not from those that face away from the spindle^16^ (Fig. 2a). This finding suggests a role for microtubules in inhibiting NE and NPC assembly^16,43^ (Fig. 2a, top, and 2b, right). Indeed, depolymerization of microtubules by nocodazole restored NPC and non-core NE assembly in an Aurora B-independent manner^16^. Consistently, the generation of micronuclei away from spindle microtubules restored NPC recruitment to lagging chromosomes and prevented the rupture of their NE^16^. Thus, a microtubule inhibition model that does not necessitate a checkpoint may explain why mitotic exit is error-prone, explaining in part the high frequency of micronuclei in cancers. It remains to be addressed how microtubules inhibit non-core NE protein deposition on lagging chromosomes. Taken together, these data show that the formation of a closed NE that lacks NPCs on lagging chromosomes is the source of the NE defects that persist into interphase^16,29^, leading to NE rupture and downstream consequences such as DNA damage.

## Micronuclei: small packages with big genomic consequences

**Fig. 3:**
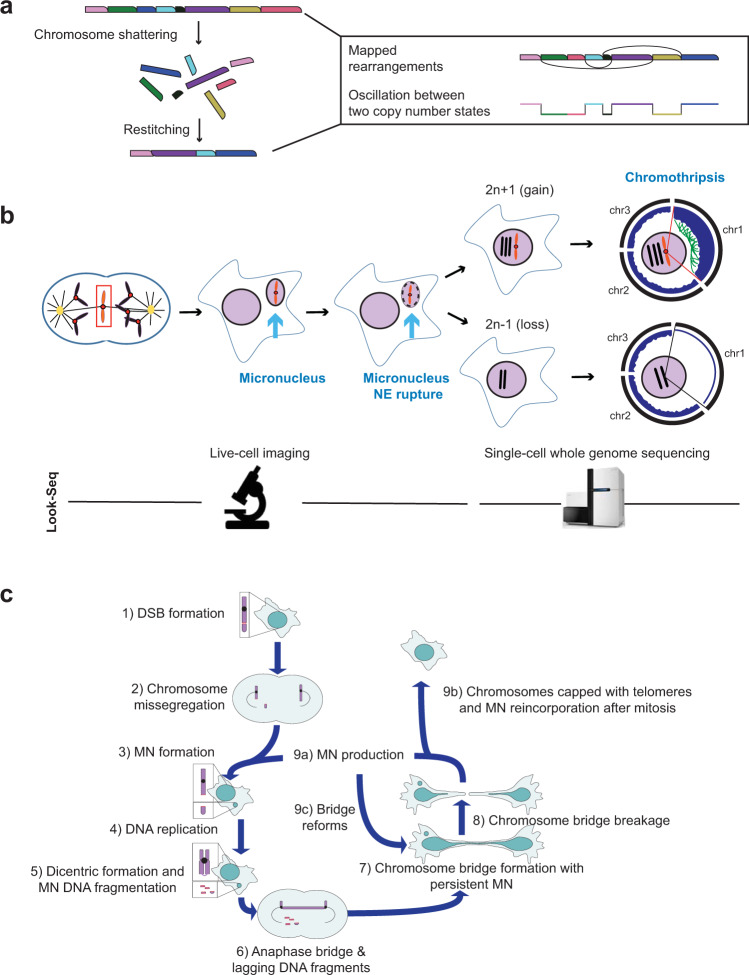
Micronuclei generate chromothripsis and ongoing genome instability. **a** Chromothripsis results from massive shattering and restitching of chromosomes in random order. Sequencing a restitched chromosome and aligning it to the reference genome reveals the formation of rearrangements (curved lines connecting rearranged segments). Pieces that are not integrated into the restitched chromosome are deleted, causing a characteristic oscillating copy number pattern. **b** The Look-Seq technique used to directly demonstrate that chromosomes contained in micronuclei undergo chromothripsis. By long-term live-cell imaging, cells were monitored for micronucleus formation, NE disruption and reincorporation of the micronucleus into a primary nucleus during the next cell division. The imaged cells were then subjected to single-cell whole genome sequencing, revealing chromothripsis on chromosomes that had been contained in micronuclei with a ruptured NE (Plot: black bars: chromosomes; blue bars: copy number; and green links: intrachromosomal rearrangements on the chromosome from the micronucleus). **c** Micronuclei (MN) can set off a cascade of instability leading to cycles of micronucleus formation and chromosome bridge formation. To escape the cycle, broken chromosomes need to be capped with telomeres, acquire a single centromere, and be reincorporated into the main nucleus. Notably, new cycles may contain only a micronucleus without a chromosome bridge or only a bridge without a micronucleus (not shown).

The discovery that DNA contained in micronuclei becomes damaged suggested that micronuclei might be a source of highly localized chromosomal rearrangements called ‘chromothripsis’^12^. Chromothripsis (Greek for ‘chromosome shattering’) is tens to hundreds of chromosomal rearrangements localized to one or a few chromosomes or chromosomal segments that occur all at once, in contrast to the gradual accrual of mutations (Figs. 1b and 3a)^44–47^. The suggestion that micronuclei might lead to chromothripsis was later affirmed, as discussed below^48–51^ (Fig. 3b).

Chromothripsis was first discovered in a patient with chronic lymphocytic leukemia^44^. This patient’s genome contained 45 genome rearrangements, 42 of which were localized to a single chromosome arm. The clustered rearrangements were joined in random orientation and order and yielded only two copy number states. The two copy number states corresponded to segments of alternating loss and maintenance of heterozygosity, or put otherwise, regions of basal copy number with interspersed deletions. This clustering of rearrangements with only two copy number states is unlikely to occur gradually but instead was predicted to occur all at once^44^. Limited microhomology at breakpoints was indicative of the rearranged chromosomes being assembled by non-homologous end-joining (NHEJ) or microhomology-mediated repair^44,51–53^. Later experiments confirmed that NHEJ factors play a major role in the repair of shattered DNA fragments from micronuclei^48^.

Similarly, complex all-at-once rearrangement patterns, termed chromoanasynthesis and chromoplexy, were also recently discovered. Whereas chromothripsis is most frequently associated with copy-number losses, chromoanasynthesis is associated with copy-number gains accompanying its rearrangements^54–56^. Chromoanasynthesis is thought to occur by template switching during DNA replication. Chromoplexy is distinct in that it consists of a closed chain of rearrangements, which can be inter- or intrachromosomal. Chromoplexy is found in many cancers and is believed to occur at replication or transcriptional hubs that bring distal segments of DNA into proximity^55,57,58^. The mechanisms that cause chromoanasynthesis and chromoplexy require further investigation.

Since its initial observation, chromothripsis has been observed in cancers, congenital disease, plant genome elimination, and even in seemingly healthy individuals^55,59^. In congenital disease it is frequently copy-number neutral, likely due to selective pressure^45^. A recent pan-cancer analysis of whole genomes found chromothripsis in at least 29% of tumors, with 100% of the liposarcomas analyzed, 85% of the osteosarcomas and 84% of the glioblastomas having at least one chromothripsis event in their genomes^46^. Additional studies observed similar trends among tumor types, with chromothripsis enriched in several tumor types, including osteosarcomas, glioblastomas, and pancreatic tumors^53,55,60,61^. Chromothripsis is causal in cancer. It is frequently clonal and causes tumor suppressor loss, fusion oncogene formation, and/or oncogene amplification by the formation of double minute chromosomes^46,55^.

In 2015, Zhang and colleagues first demonstrated that micronuclei cause chromothripsis^51^. Using ‘Look-Seq’, a novel technique of tracking cells containing micronuclei and subjecting their progeny to single-cell sequencing (Fig. 3b), they showed that the chromosome contained in a micronucleus exhibited several hallmarks of chromothripsis^44,51^. First, there was a massive enrichment of rearrangements on the chromosome contained in the micronucleus compared to the remainder of the genome. Second, some chromosomes from micronuclei segregated to a single daughter (Fig. 3b, top). These were dominated by copy-number neutral rearrangements like those observed in congenital chromothripsis. In contrast, some chromosomes were shattered and their fragments were passed independently to both daughters. In these cases, DNA segments that segregated to one daughter manifested as deletions in the other daughter (Fig. 3b), producing the characteristic oscillating two-copy number state of chromothripsis (Fig. 3a, b). Together with other recent work using micronuclei whose contents were known a priori, it is now clear that micronuclei generate chromothripsis at remarkably high rates, regardless of whether the bulk of the micronucleus is reincorporated into the primary nucleus^48–51^.

The lack of requirement of bulk reincorporation is surprising, as it was thought that fragments needed to be reincorporated into a repair-competent primary nucleus for rearrangement to occur^48,50,62^. However, in these cases, it is possible that some fragments not visible by microscopy are reincorporated and repaired. Alternatively, aberrant mitotic DNA synthesis might cause rearrangement without reincorporation into the main nucleus when a NE is not present^63,64^.

Although it is clear that micronuclei are at least one source for chromothripsis, it is unknown exactly how the DNA in micronuclei is shattered. Delayed replication timing in micronuclei has led to speculation that the DNA contained in micronuclei might be shattered by premature mitotic entry^11,12,48^. In support of this, premature chromosome condensation in G2 cells induced by treatment with the phosphatase inhibitor calyculin A caused fragmentation of chromosomes targeted to micronuclei^48^.

Alternatively, it has been proposed that exposure to cytoplasmic nucleases upon NE disruption could cause DNA damage in micronuclei. One candidate nuclease is the 3’ exonuclease TREX1, which has been observed at ruptured micronuclei^65^. In support of a role for TREX1 in chromothriptic rearrangement, TREX1-deficient single-cell clones harbored fewer highly complex rearrangements after telomere crisis and chromosome bridge formation than TREX1-intact clones^65,66^. On the other hand, another group did not see a reduction in complex rearrangements when sequencing TREX1-deficient single-cells after bridge formation, suggesting that an alternative mechanism for chromosome shattering might exist^64^. If TREX1 is in fact the nuclease responsible for complex chromosome breakage, several unknowns remain. Because TREX1 is an exonuclease it requires the presence of nicks as a substrate for activity. The source(s) of these nicks remains unknown, although the ribonuclease RNaseH2 is one candidate^65,67^. It is also unknown how TREX1 would cause the formation of double-strand breaks. To produce a double-strand break, the exonuclease would need to collide with a nick on the opposite strand or would require the collision of two TREX1 exonuclease tracts on opposite strands^65^. Additional enzymes that could be responsible for generating chromothripsis include topoisomerase IIb, which has been observed in areas of torsional stress induced by membrane distortion^68^, and abasic endonucleases, such as APE1, an enzyme that is active during the repair of deaminated bases after APOBEC activity^65^.

Although micronuclei are a major cause of chromothripsis, chromosome bridges have also been shown to result in chromothripsis^63,65,66^. The extent to which micronuclei contribute to genome instability compared to other nuclear atypia, especially in the context of cancers, is worthy of further exploration. Notably, micronuclei and bridge formation frequently follow one another during tumor development^64,69,70^. One possible explanation for this sequence is that micronuclei arising from DNA double-strand breaks leave behind the uncapped centromere-containing part of the chromosome, which is then susceptible to improper repair and chromosome bridge formation^50^. Chromosome bridges can then undergo cycles of instability, including the formation of new micronuclei^64,65,69,71^. Therefore, DNA in micronuclei might just be the start of a cascade of genome instability (Fig. 3c).

## Raising the alarm: micronuclei activate cytoplasmic DNA-sensing and innate immune signaling

In addition to causing DNA damage, the presence of DNA from ruptured micronuclei in the cytosol signals the activation of the cGAS-STING pathway of innate immunity^17–19,72,73^ (Fig. 1b). Whereas self-DNA normally resides in the nucleus and is thus largely immune from detection, foreign DNA residing in the cytosol upon pathogen infection or micronucleus envelope disruption can be recognized by cyclic GMP-AMP synthase (cGAS). The cytoplasmic DNA-sensing cGAS-STING pathway thus plays a canonical role in antiviral innate immunity. Upon dsDNA binding by cGAS, 2’-5’-cGAMP is synthesized, leading to the activation of stimulator of interferon genes (STING). Through downstream signaling cascades, STING then induces the transcription and secretion of type I interferon and inflammatory cytokines^72,73^.

The work of various groups has demonstrated that self-DNA from DNA damage-induced micronuclei with ruptured NE can act as a trigger for the cGAS-STING pathway^17,18^ (Fig. 1b, right). By utilizing single-cell RNA sequencing and light microscopy, Mackenzie et al. showed that interferon-stimulated genes downstream of cGAS are upregulated in cells containing micronuclei^18^. This inflammatory response was shown to be dependent upon the presence of micronuclei and not the DNA damage used to cause micronucleus formation per se^17,18^. Furthermore, cells with ongoing chromosomal instability contain cGAS-positive micronuclei and undergo a cGAS-STING-dependent proinflammatory response^19^.

Other self-DNA triggers of cytoplasmic DNA-sensing innate immunity are cytoplasmic chromatin fragments (CCFs) that arise under various cellular stresses (Fig. 1b, right). For example, aneuploidy-induced senescence is likely to be accompanied by rupture-prone micronuclei from chromosome missegregation^19,74^. The contents of micronuclei, if not completely reincorporated into the main nucleus in the following cell cycle, may become CCFs. Furthermore, several studies have reported that cells undergoing senescence due to oncogenic, genotoxic, or oxidative stress show cGAS-STING-dependent inflammatory responses via cytoplasmic DNA^75–77^. Cellular senescence is characterized by terminal cell cycle arrest accompanied by the characteristic expression and secretion of proinflammatory cytokines, collectively termed the senescence-associated secretory phenotype (SASP)^78^. During senescence, CCFs originate from autophagic degradation of nuclear lamina^79,80^. Consistent with this, inhibition of autophagy attenuates lamin B1 degradation, causing a reduction in the generation of CCFs and senescence^79^. Moreover, genetic perturbation of the cGAS-STING pathway under these stress conditions protects cells from senescence and suppresses the SASP program, suggesting a role for CCFs in self-DNA recognition, SASP, and senescence^75–77^. Alternatively, self-DNA and subsequent cGAS-STING activation stimulates a different pathway, causing senescence-independent cell death, as demonstrated in cells undergoing replicative stress after telomere crisis^81^.

Although CCFs and micronuclei appear to be generated by different mechanisms, CCFs and micronuclei share many features, including the accumulation of markers for DNA damage (as determined by γH2AX), inactive transcription (as determined by heterochromatic histone mark H3K27me3), and NE disruption (as determined lamin B loss)^15,16,80^. Thus, further analysis would better define the sources of cytoplasmic self-DNA and the relative contribution of CCFs, micronuclei or mitochondrial DNA^72,73,82^ in cGAS-STING activation during cellular senescence in different contexts.

## The two faces of cytoplasmic DNA-mediated innate immune activation in cancer

The consequences of cytoplasmic DNA sensing and activation of the innate immune pathway are complex and context dependent. Self-DNA recognition and subsequent cGAS-STING-mediated inflammation present a double-edged sword: they can suppress tumorigenesis but also might promote it.

The former was demonstrated from a surprising observation: abscopal effect, the long known phenomenon wherein local irradiation suppresses tumors far from the site of irradiation, was revealed to be STING-dependent in mice^17^. Because irradiation induces micronucleation and NE rupture, this observation suggested that the antitumoral effect of innate immune pathway activation during abscopal effect might be elicited by DNA from micronuclei. In support of this, it is known that activation of the cytoplasmic DNA sensing pathway can induce both autocrine and paracrine signaling effects. This occurs because cGAMP can diffuse through gap junctions into neighboring cells^83^, and cGAMP and downstream SASP factors can be secreted^78,84,85^. Thus, only a few cells with ruptured micronuclei might activate immune signaling in a large amount of tissue, making micronuclei an appealing candidate source for the abscopal effect.

Tumor suppressive roles of self-DNA recognition are also evident in senescent cells. For example, cells undergoing repeated chromosome segregation errors not only permanently arrest in the cell cycle and undergo senescence, but are also cleared by immune cells, such as natural killer (NK) cells^74^. Immune clearance of chromosomally unstable cells might be caused by the activation of self-DNA recognition pathways and subsequent SASP, which together can shape the immune landscape in a manner similar to that of the immune clearance of cancer cells^86^. Consistent with this idea, in cells undergoing senescence in response to oncogenic stress (by oncogenic NRasV12 expression), CCF-mediated STING activation is indeed required for the clearance of senescent cells and the suppression of tumorigenesis^77^. These results suggest that boosting cGAS-STING innate immunity and exploiting NE defects can be an effective cancer immunotherapy strategy for immune clearance of cancer or precancerous cells.

Beyond the antitumoral function of innate immunity, cytoplasmic DNA sensing by the cGAS-STING pathway has also emerged as a mechanism that promotes inflammation-driven tumorigenesis^19,87^. For example, cells displaying high chromosomal instability with ongoing chromosome segregation errors and ruptured micronuclei have been reported to promote cellular metastasis by chronic activation of the cGAS-STING pathway^19^. Therefore, further elucidation of what switches cGAS-STING signaling between tumor prevention and activation will be important if this pathway is to be used therapeutically. To this end, investigation will be required to dissect the molecular nature of cGAS-STING downstream signaling and the composition of SASP factors that dictate different outcomes in tumor promotion and suppression.

## Perspectives and conclusions

Recent studies have advanced our understanding of many processes in micronucleus biology with broad impacts in both basic research and clinical fields. The important basic question as to how chromosome segregation is coordinated with NE assembly at the end of mitosis has begun to be addressed. In answering this question, studies have shown how the normal NE assembly process proceeds and have revealed the origin of the NE defects in micronuclei that lead to NE rupture. It has also become clear that micronuclei are not simple consequences of tumors, but instead are main contributors to tumorigenesis. The loss of NE integrity of micronuclei has been intricately associated with chromothripsis, many defects in cellular function, and the activation of innate immune signaling. By using new tools and approaches including single-cell whole genome and RNA sequencing combined with imaging to trace the history of nuclear aberration, and bulk DNA and RNA sequencing of cells containing micronuclei, work over the past decade has defined how chromothripsis arises, how and why the NE of micronuclei is prone to rupture, and how a fragile NE causes innate immune activation.

Many important questions remain unanswered in the study of micronuclei and related phenomena. For example, the mechanism by which spindle microtubules and/or spindle geometry restrict NPC assembly of micronuclei and thus generate a fragile NE remains to be explored. In addition, the mechanisms of DNA damage in micronuclei remain unknown. In particular, which enzymes, if any, are responsible for chromosome fragmentation during chromothripsis? Further studies as to how different sources of self-DNA, including micronuclei, CCFs, and mitochondrial DNA, contribute to cGAS activation would help to better understand the innate immune response. Additionally, a deeper understanding of the context-dependent consequences of innate immune activation by NE rupture or cytoplasmic DNA will help develop cancer immunotherapy strategies. Finally, finding predictive biomarkers of abnormal nuclear structures, including ruptured micronuclei and CCFs in cultured cells, tissues and in vivo, would help to define patients who will benefit from therapeutic strategies that exploit NE integrity and the cGAS-STING innate immune pathway.
